# Liquid Biopsy of Circulating Tumor Cells and DNA in the Context of PSMA Radiopharmaceutical Therapy

**DOI:** 10.2967/jnumed.125.270651

**Published:** 2026-01

**Authors:** Adrien Holzgreve, Christine E. Mona, Koichiro Kimura, Xinmin Li, Yazhen Zhu, Hsian-Rong Tseng, John K. Lee, Alexandra Drakaki, Isla P. Garraway, Matthew B. Rettig, Jeremie Calais, Ali Salavati

**Affiliations:** 1 Ahmanson Translational Theranostics Division, Department of Molecular and Medical Pharmacology, David Geffen School of Medicine, UCLA, Los Angeles, California;; 2 Department of Nuclear Medicine, LMU University Hospital, LMU Munich, Munich, Germany;; 3 Jonsson Comprehensive Cancer Center, UCLA, Los Angeles, California;; 4 Department of Pathology and Laboratory Medicine, UCLA Technology Center for Genomics and Bioinformatics, Los Angeles, California;; 5 California NanoSystems Institute, Crump Institute for Molecular Imaging, Department of Molecular and Medical Pharmacology, UCLA, Los Angeles, California;; 6 Division of Hematology and Oncology, Department of Medicine, David Geffen School of Medicine, UCLA, Los Angeles, California;; 7 Molecular Biology Institute, UCLA, Los Angeles, California;; 8 Department of Urology, David Geffen School of Medicine, UCLA, Los Angeles, California;; 9 Department of Surgical and Perioperative Care, VA Greater Los Angeles Healthcare System, Los Angeles, California; and; 10 Department of Medicine, VA Greater Los Angeles Healthcare System, Los Angeles, California

The concept of “liquid biopsy” has gained high interest in oncology and is being studied more as it is gradually getting incorporated in patient care for multiple cancer types. Liquid biopsy describes the analysis of circulating tumor cells (CTCs) or smaller pieces of cancer cells such as circulating tumor DNA (ctDNA) and cell-free DNA that have been released into body fluids, for example into circulation. Although tumor biopsy is the gold standard in cancer diagnosis, liquid biopsy allows for serial sampling that gives the opportunity for profiling of genetic and molecular changes taking place in a tumor at different time points during treatment or monitoring of the disease. Other potential applications include but are not limited to initial indication of cancer diagnosis, disease recurrence, assessment of drug resistance, and monitoring of treatment response. Although only 7 oncology liquid biopsy trials were reported to have been initiated in 2007, the number had grown to approximately 200 trials by 2022, correlating with the increased number of targeted drugs in development and the lower cost of sequencing (1).

Radiopharmaceutical therapy (RPT) targeting prostate-specific membrane antigen (PSMA) has been increasingly used in clinical practice and trials since its approval for patients with metastatic castration-resistant prostate cancer (mCRPC) in 2022. Liquid biopsy is evolving as a promising addition in the evaluation of PSMA RPT, as preliminary data have provided the rationale for further investigation.

In a prospective proof-of-principle study of 20 patients treated with [^177^Lu]Lu-PSMA-617, 20% of CTC samples showed no PSMA expression, despite unequivocal PSMA expression of solid metastases in PET (2). A high fraction of PSMA-negative CTCs was linked to both shorter overall and shorter progression-free survival (2). Pretreatment ctDNA findings in a cohort of 44 patients indicated that gene amplifications, particularly in *FGFR1* (encoding fibroblast growth factor receptor 1) and *CCNE1* (encoding cyclin E1) may serve as biomarkers for resistance to PSMA RPT (3), although these amplifications are not typically observed in tumor tissue of patients with mCRPC. In an analysis of cell-free DNA from blood samples of 46 patients who started PSMA RPT, patients with genetic alterations in the androgen receptor gene or *PI3K* pathway genes did not experience a long-lasting benefit from PSMA RPT (4). A study examining ctDNA in 32 men treated with PSMA RPT found shorter prostate-specific antigen response, progression-free survival, and overall survival to be associated with alterations in aggressive variant prostate cancer genes (*TP53*, *RB1*, or *PTEN*) (5). First results for larger trials with standardized patient populations, audited outcomes, and randomizations are available, giving the opportunity to more robustly examine the potential of biomarkers. A subanalysis using data from 180 patients in the TheraP trial, a randomized phase 2 study of PSMA RPT versus cabazitaxel in mCRPC, revealed lower ctDNA fraction to be associated with higher odds for response to PSMA RPT compared with cabazitaxel, and that the predictive value of the ctDNA fraction was additive to PSMA PET imaging parameters (6). Subanalyses of the PSMAfore trial, a randomized phase 3 study comparing PSMA RPT to a switch in androgen receptor pathway inhibitor in the prechemotherapy mCRPC setting, showed that both a higher ctDNA fraction at baseline and ctDNA fraction dynamics until cycle 2, as well as androgen receptor gene, *TP53*, and *PTEN* alterations, were associated with shorter survival also in taxane-naïve patients treated with PSMA RPT (7,8).

Although these preliminary data suggest prognostic and predictive values of liquid biopsy biomarkers regarding PSMA RPT, mainly suggesting that high ctDNA fraction or CTC count in general may portend poor or short response, more data are needed to substantiate a clinical role of liquid biopsy in this setting. Implementation into clinical trials in principle seems feasible, and potential applications are numerous (summarized in Fig. 1). First, peripheral blood tumor biomarkers may be assessed at baseline before PSMA RPT to enhance PET-based imaging biomarkers and to narrow the suitability criteria, with an aim to refine patient selection for PSMA RPT. Further, they have the capability to monitor treatment response and alterations in tumor tissue linked to treatment resistance over time, with a dwindling role for numeric CTC measurements toward leveraging more advanced methods, such as profiling nucleosome footprints in ctDNA (9). Beyond the correlative analyses performed thus far, biomarker-driven clinical trials may facilitate the generation of evidence for the clinical use of liquid biopsy in the context of PSMA RPT. This may contribute to more precise patient management, as it can be performed more frequently than PSMA-directed imaging at a lower cost and combined with other markers, such as prostate-specific antigen, with the ultimate goal to direct the optimal imaging time points. Noteworthy, the underlying pathophysiologic bases for response versus treatment resistance to PSMA RPT are not well understood. Hence, liquid biopsies may contribute to the analysis of the biologic and molecular determinants of treatment resistance to PSMA RPT, such as the genomic aberrations in DNA-repair genes, without the need for multiple invasive tumor biopsies, whereas imaging can provide complementary information, such as the spatial heterogeneity of PSMA expression over time (2,10). The depiction of molecular alterations in tumor tissue with PSMA RPT may also enable the identification of future therapeutic targets when patients’ disease is progressing with RPT. Subsequently, this could lead to a combination of theranostic approaches, such as the cotargeting of heterogeneous tumors or a complete change in therapeutic agent. Although the field of liquid biopsy as a companion to nuclear medicine applications is still in its early stages, we expect that it will continue to be incorporated, with the ultimate goal of improving medical clinical decision-making and enhancing patient care.

**FIGURE 1.**
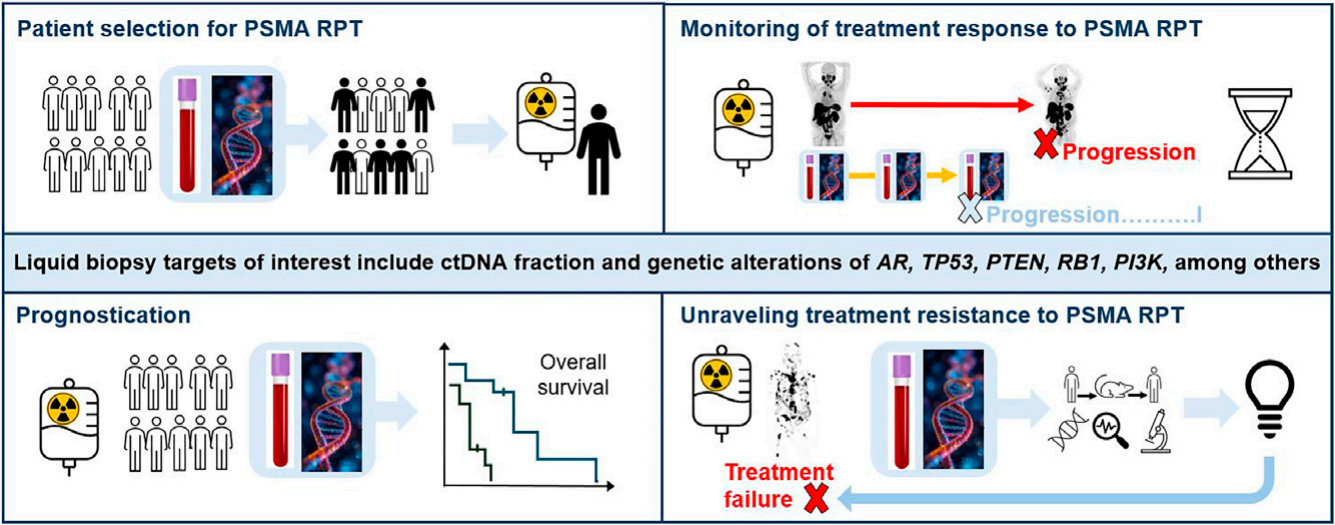
Overview of potential liquid biopsy applications and genetic targets in context of PSMA RPT.

## DISCLOSURE

Adrien Holzgreve is funded by the Deutsche Forschungsgemeinschaft (DFG, German Research Foundation; 545058105). He reports fees from ABX advanced biochemical compounds outside the submitted work. Matthew Rettig reports personal fees (consultant and speakers’ bureau) from Bayer and Johnson & Johnson, consulting fees from Immune Bio, and grant support from Pfizer, Lantheus, Novartis, Merck, and Johnson & Johnson. Johannes Czernin is the recipient of grants from the Prostate Cancer Foundation (2022 Tactical Award, TACT01; 2019 Challenge Award, 19CHAL09; 2017 Challenge Award, 17CHAL02) and the Jonsson Comprehensive Cancer Center NIH National Cancer Institute Cancer Center Support Grant (P30 CA016042). Johannes Czernin and Christine Mona are recipients of a grant from the NIH (R01CA279094). Johannes Czernin is a founder of SOFIE Biosciences and Infinity TopCo and holds equity in the company and in intellectual property invented by him, patented by the University of California, and licensed to SOFIE Biosciences. He serves as scientific advisor for SOFIE biosciences. He is a founder of Trethera Therapeutics and holds equity in the company and in intellectual property invented by him, patented by the University of California, and licensed to Trethera. He serves on the scientific advisory board of Aktis Oncology. Jeremie Calais was the recipient of grants from the Prostate Cancer Foundation (2020 Young Investigator Award; 20YOUN05), the Society of Nuclear Medicine and Molecular Imaging (2019 Molecular Imaging Research Grant for Junior Academic Faculty), the Philippe Foundation Inc. (New York), and the ARC Foundation (France; International Mobility Award SAE20160604150). He reports grants from Progenics for PyL Research Access Program; investigator-initiated trial NCT04457245; personal fees (consultant) from POINT Biopharma, Curium Pharma, GE HealthCare, Blue Earth Diagnostics, Janssen Pharmaceuticals, and Progenics; personal fees (blinded independent central reader) from Advanced Accelerator Applications, Radiomedix, Progenics, and Exini; and personal fees (speaker fees) from IBA RadioPharma and Telix Pharmaceuticals outside the submitted work. No other potential conflict of interest relevant to this article was reported.
